# Recent Advancements in the Diagnosis, Prevention, and Prospective Drug Therapy of COVID-19

**DOI:** 10.3389/fpubh.2020.00384

**Published:** 2020-07-10

**Authors:** Waquar Ahsan, Hassan A. Alhazmi, Kuldeep Singh Patel, Bharti Mangla, Mohammed Al Bratty, Shamama Javed, Asim Najmi, Muhammad Hadi Sultan, Hafiz A. Makeen, Asaad Khalid, Syam Mohan, Manal M. E. Taha, Shahnaz Sultana

**Affiliations:** ^1^Department of Pharmaceutical Chemistry, College of Pharmacy, Jazan University, Jazan, Saudi Arabia; ^2^Substance Abuse and Toxicology Research Centre, Jazan University, Jazan, Saudi Arabia; ^3^Department of Pharmacy, NRI Institute of Research & Technology, Bhopal, India; ^4^Department of Pharmaceutics, School of Pharmaceutical Education and Research, Jamia Hamdard University, New Delhi, India; ^5^Department of Pharmaceutics, College of Pharmacy, Jazan University, Jazan, Saudi Arabia; ^6^Department Clinical Pharmacy, College of Pharmacy, Jazan University, Jazan, Saudi Arabia; ^7^Department of Pharmacognosy, College of Pharmacy, Jazan University, Jazan, Saudi Arabia

**Keywords:** 2019-nCoV, SARS-CoV-2, coronavirus, COVID-19, diagnosis, vaccines, clinical trials, drug repurposing

## Abstract

Severe acute respiratory syndrome coronavirus (CoV)-2 (SARS-CoV-2), previously called 2019 novel CoV, emerged from China in late December 2019. This virus causes CoV disease-19 (COVID-19), which has been proven a global pandemic leading to a major outbreak. As of June 19, 2020, the data from the World Health Organization (WHO) showed more than 8.7 million confirmed cases in over 200 countries/regions. The WHO has declared COVID-19 as the sixth public health emergency of international concern on January 30, 2020. CoVs cause illnesses that range in severity from the common cold to severe respiratory illnesses and death. Nevertheless, with technological advances and imperative lessons gained from prior outbreaks, humankind is better outfitted to deal with the latest emerging group of CoVs. Studies on the development of *in vitro* diagnostic tests, vaccines, and drug re-purposing are being carried out in this field. Currently, no approved treatment is available for SARS-CoV-2 given the lack of evidence. The results from preliminary clinical trials have been mixed as far as improvement in the clinical condition and reduction in the duration of treatment are concerned. A number of new clinical trials are currently in progress to test the efficacy and safety of various approved drugs. This review focuses on recent advancements in the field of development of diagnostic tests, vaccines, and treatment approaches for COVID-19.

## Introduction

In late December 2019, an outburst of a mysterious disease, which was regarded as pneumonia of unknown cause, appeared in Wuhan city, Hubei Province, China. It was later identified to be caused by a novel coronavirus (CoV) known as 2019-novel CoV (2019-nCoV), which was not observed previously in humans nor animals (1–3). The disease caused by 2019-nCoV is highly contagious and 8,735,721 cases have been confirmed as of June 19, 2020 (22:30 GMT), with 461,519 deaths reported in more than 200 countries (4). The pathogen was momentarily named severe acute respiratory syndrome CoV 2 (SARS-CoV-2), and the pertinent contaminated condition was termed CoV disease 2019 (COVID-19) by the World Health Organization (WHO). Initially, the majority of confirmed cases of COVID-19 were linked to Huanan seafood market of Wuhan city, which was closed on January 1, 2020. COVID-19 has increased at a considerable rapid rate and is now affecting almost all countries of the world; the outbreak was affirmed as a worldwide pandemic by WHO on March 11, 2020 (5).

CoVs are a highly varied cluster of positive-sense, enveloped single-stranded RNA viruses (6, 7). They cause numerous diseases concerning respiratory, hepatic, neurological, and enteric systems of varied severity amongst humans and animals (8, 9). Other human CoVs, including HCoV-OC43, HCoV-229E, HCoV-NL63, and HCoV-HKU1, cause a low incidence of respiratory infections and mild illness (10, 11). However, in the past several years, two deadly strains of CoVs, namely, Middle-East respiratory syndrome CoV (MERS-CoV) and SARS-CoV have emerged, causing severe infections in humans (12, 13). Throughout the SARS-CoV epidemic, over 8,000 people were infected globally, resulting in around 800 mortalities and a 10% mortality rate. Similarly, 857 official cases were reported for MERS-CoV, with 334 deaths and a mortality rate of around 35% (14, 15). The seventh member of CoV family infecting humans is the novel SARS-CoV-2, which is currently the most contagious CoV. The major symptoms of COVID-19 include high-grade fever, dry cough and fatigue, which are analogous to those of MERS-CoV and SARS-CoV-related infections. Various discrete and overlapping features are related to the pathogenesis and subsequent pathology of the CoVs that cause severe infections in humans (16). Published studies reported the remedial aspects, pathology, radiology, and virology of COVID-19. However, reviews covering recent developments in the field are scarce. The rationale of this review is to cover important aspects of the pathogen causing COVID-19, its clinical features, diagnosis, and treatment efforts, which were developed with the pathology and epidemiology on the basis of existing evidences. Most of the recent advancements in the development of *in vitro* diagnostic tests (IVD) were collected from the websites of corresponding manufacturers, including those which obtained the Emergency Use Authorization (EUA) by the Food and Drug Administration (FDA). The data on completed and ongoing clinical trials in various countries were collected from authentic sources, such as published articles, preprints servers, National Institute of Health and U.S. National Library of Medicine, and their important findings were summarized and discussed.

## CoV Pathogen

The causative pathogen of COVID-19 is 2019-nCoV, which was first detected in January 2020 and later termed as SARS-CoV-2 (17, 18). This pathogen is a single-stranded RNA virus (19) that probably originated from bats owing to its similar genetic sequence to other CoVs (7, 20). Although SARS-CoV-2 shares genetic features attuned with the other members of the CoV family, it possesses considerably varied genetic sequence compared with that of earlier sequenced CoVs. SARS-CoV-2 shares around 79.5% identical genetic sequence with SARS-CoV and 96.2% genetic sequence similarity with RaTG13, a short RNA-dependent RNA polymerase (RdRp) region present in the CoV that originated from bats. SARS-CoV-2 belongs to the genus *Beta coronavirus* and subgenus *Sarbeco* virus and is different from SARS-CoV (21, 22). SARS-CoV-2 first originated in bats with pangolins as an intermediate mammalian host (23, 24). A closely related virus obtained from the lung samples of Malayan pangolin showed similarity with the SARS-CoV-2 given that SARS-CoV-2 and Pangolin-CoV share five key amino-acid substitutions in the receptor binding domain (RBD) and are 91.02% identical. Pangolin-CoV is the second closest to SARS-CoV-2 after RaTG13.

**Figure 1:**
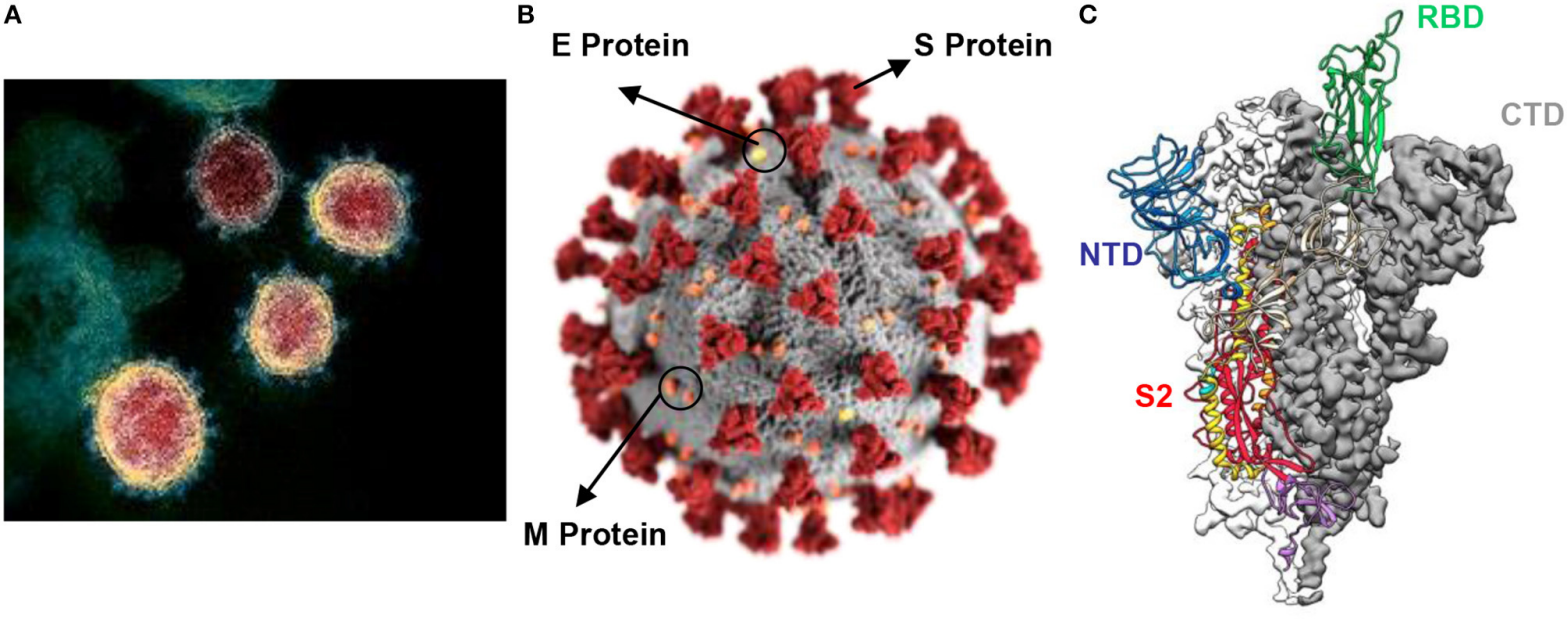
**(A)** Electron micrograph of SARS-CoV-2 virions; **(B)** illustration of the virion showing presence of S protein, E protein and M protein at the surface; **(C)** atomic-level trimeric ectodomain of SARS-CoV-2 spike (S) protein showing S2 subunit, receptor binding domain (RBD), N-terminal domain (NTD), and C-terminal domain (CTD) [image source: U.S. National Institute of Allergy and Infectious Diseases (NIAID-RML) and is available for reproduction for research purposes].

Figures 1A–C illustrates the electron micrograph of virions along with the three-dimensional structure of its spike (S) protein. The envelope (E) S protein is used by the CoV to attach to the host cell (25). The S protein is responsible for binding to the receptor and host membrane (M) fusion and is vital for the determination of transmission capacity and tropism of hosts (26–28). The two functional domains of S protein are regarded as S1 (liable for binding to receptor) and S2 (assists in cell M fusion) (29). Three-dimensional structural analysis of the virions revealed the presence of RBD (Figure 1C), which consists of an external subdomain and a core and can bind to angiotensin-converting enzyme II (ACE2) receptors in a manner similar to that of SARS-CoV (21, 22, 25). The crystal structure of SARS-CoV-2 S protein C-terminal domain in complex with human ACE-2 was developed, revealing the strong affinity of C-terminal domain with ACE-2 with high number of atomic contact points (30). Two additional crystal structures of SARS-CoV-2 RBD bound to ACE-2 were reported (31, 32). The residues of SARS-CoV-2 RBD, which are critical in binding to ACE-2, were identified. Surface plasmon resonance was employed to show that SARS-CoV-2 RBD binds more strongly to ACE-2 than SARS-CoV (32).

The S protein is the primary target for vaccines and neutralizing antibodies, whereas the S1 subunit acts as the most vulnerable antigen causing immunogenicity. In addition to S protein, SARS-CoV-2 has a nucleocapsid (N) protein containing viral RNA, which is commonly detected by the immunoassay of blood and serum samples of infected patients during the early days of infection. M protein is the most abundant protein in the virus, whereas the pathogenesis is attributed to the E protein. SARS-CoV-2 enters the host cells in the respiratory system by binding to the ACE2 receptor and multiplies rapidly to form new virions. The signs and symptoms of the disease generally appear after 2–14 days of the infection, that is, the viral incubation period.

## Clinical Features

Most of the COVID-19 patients are aged 30–79 years old with a mean range of 49–59 years [34, 35], and relatively fewer cases are reported in children below 15 years. Male patients constitute more than half of the reported cases, including those with one or more coexisting medical complications, such as diabetes, hypertension, cardiovascular disorders, or cancer (33, 34). The focal symptoms of COVID-19 include dry cough, fever, myalgia, fatigue, and dyspnoea, whereas the scarce symptoms include headache, increased sputum, diarrhea, and haemoptysis. In patients with severe and critical case of COVID-19, the viral pneumonia progresses into acute respiratory distress syndrome (ARDS) and multi-organ system failure accompanied by cytokine storm. Patients requiring intensive care unit admission due to hypoxemic respiratory failure suffer mainly from ARDS and are placed on mechanical ventilation; a high rate (50%) of mortality is observed in patients on mechanical ventilation (35).

In 7–27.8% of the patients suffering from COVID-19, troponin levels are elevated and may be implicated in cardiovascular disorders, such as type I myocardial infarction, decompensated heart failure or arrhythmia (36). Elevated troponin levels also occur in patients with ARDS, intense activation of inflammatory cytokines, hypercoagulability, and myocarditis. Most of COVID-19 patients experience mild to moderate symptoms and recover with standard care without any special intervention. However, old-age patients and people with other underlying major conditions are susceptible to serious illnesses. Nevertheless, the most effective way to prevent transmission is sufficient awareness and preparedness for the virus. Many patients with viral infection are asymptomatic and are the most frequent carrier of the disease, thus contributing to the major spread of infection. Therefore, the identification of infected patients is the first and most important step to combat SARS-CoV-2 infection. The WHO has also stressed that all countries must employ intensive diagnostic measures to as much cases as possible to identify and quarantine the infected persons to avoid the further spread of infection.

## Development of IVD Tests

Thus far, the WHO has approved three techniques for the detection of CoVs. In the reverse transcription-polymerase chain reaction (RT-PCR) assay for SARS virus, clinical specimens from nasopharyngeal or oropharyngeal samples are obtained, and three genes are targeted. These targets include the Orf1b gene (human RNA polymerase protein), N-gene (N protein), and the E-gene (E protein) (37). Several PCR kits based on RT-PCR or quantitative RT-PCR methods are available in the market. The immunoassay using enzyme-linked immunosorbent assay (ELISA) is less costly but is also less sensitive than PCR. SARS-CoV-2 is confirmed by the presence of immunoglobulin (Ig) G in immunofluorescence assay (IFA) (38). These tests are performed on the blood samples of the infected patient and by testing for a specific antibody that is produced by the body as part of its defense mechanism. The third method is the laboratory isolation and culture of virus from any specimen. However, this method is a long procedure and requires confirmation by PCR.

### Serological Tests

Serological tests are blood-based tests that identify whether the tested person is exposed to an infection. These tests are based on the presence of antibodies for a particular pathogen acting as antigens. These antigens are recognized by the immune system of the infected person as foreign bodies and develop specific antibodies to fight the infection. Given that SARS-CoV-2 is a novel virus, and the antibodies developed by the immune system of infected people are specific and only present in people with COVID-19, these antibodies can act as markers for the disease. These tests are specifically useful in identifying people who had the infection and have subsequently recovered from it, thus providing data on the actual prevalence of the disease. Serological tests are of various types, including neutralization tests, IFA, ELISA and Western Blotting.

Serological tests rely on the presence of IgM and IgG antibodies present in the body of infected patients. Both antibodies act as biomarkers of diseases and are detected by immunoassay techniques. In general, these antibodies are produced after the second week of SARS-CoV-2 infection and are effective only after such period. IgM antibodies can be detected after 10–30 days of infection, whereas IgG is expressed after 20 days of infection (39). IgM antibodies are produced earlier than IgG, but they disappear within several days. Meanwhile, the IgG antibodies last for a long period, giving protection against the disease. Serological tests are often coupled with RT-PCR based on the presence of viral RNA. The combination of both techniques increases the sensitivity of detection and yields confirmatory results. A recently published study in the pre-print server revealed the development of specific serological tests which utilize serological enzyme-like ELISA; these tests detect antibodies to clone the SARS-CoV-2 S-protein, its RBD and the N protein (40).

Serological tests for use by authorized laboratories are approved by FDA through EUA. To date, a number of serological tests have been granted authorization under EUA. In May this year, FDA has approved an anti-SARS-CoV-2 ELISA test kit, which is based on the detection of IgG antibodies, developed by Euroimmun US Inc., NJ (41). This test detects IgG antibodies in human serum and plasma (K^+^-ethylenediaminetetraacetic acid (EDTA), Li^+^-heparin and Na^+^-citrate). Elecsys Anti-SARS-CoV-2 is another serological test authorized by FDA (Roche Diagnostics, IN) (42). This test can detect the antibodies specific to SARS-CoV-2 are present in serum and plasma (EDTA or heparin). The SARS-CoV-2-specific antigens are immobilized on streptavidin-coated microparticles, and the antigen–antibody complexation is detected by electrochemiluminescence by using special analysers. FDA have recently authorized other serological tests through EUA; these tests include New York SARS-CoV Microsphere Immunoassay for Antibody Detection (New York State Department of Health, NY) (43), Platelia SARS-CoV-2 Total Ab Assay (Bio-Rad Laboratories, WA) (44), SARS-CoV-2 IgG assay (Abbott Laboratories Inc., IL) (45), and LIAISON SARS-CoV-2 S1/S2 IgG device (DiaSorin Inc., MN) (46).

### Point-of-Care Testing (POCT)

Several POCT or rapid diagnostic tests for immunodiagnostic detection of SARS-CoV-2 have been developed. However, the WHO recommends the use of these tests in research settings only. These tests should not be used for clinical decision-making settings unless other specific indications are present. These POCT methods are very rapid and can give results within several minutes; however, they can only detect actively replicating viruses and can be used for the identification of acute or early infection only (47–50). Vivalytic COVID-19 detection kit is POCT-based diagnostic test developed by Bosch, Germany in collaboration with Randox Laboratories, UK (51). This kit can detect SARS-CoV-2 along with nine other respiratory viruses. This fully automated POC molecular testing device uses samples obtained from the nose or throat and placed in cartridges containing the required reagents. The cartridge is then placed in a Vivalyte analyser to determine the results.

Similarly, in the field of rapid detection assays, Abbott ID Now™ COVID-19 test assay can detect antigens in <5 min (52, 53). This molecular POCT uses isothermal nucleic-acid-amplification technology to specifically detect SARS-CoV-2 RNA. The added advantage of this technique is its portability and lightweightness, which enable its smooth transport to different locations. Other rapid lateral-flow immunoassay (LFIA)-based POCTs have also been developed recently; they detect IgG and IgM antibodies, which are expressed due to SARS-CoV-2 infection, in suspected people. BioMedics, USA developed a POCT that detects antibodies within 10 min (54). This technique also utilizes microliter amount (20 μL) of serum or plasma from the patient and can be used at any location without any skilled technique. The LFIA-based rapid test developed by Cellex Inc., USA was also approved by FDA for EUA (55). This rapid test detects IgM and IgG antibodies binding to SARS-CoV-2 N protein, with a sensitivity of 93.8% and specificity of 95.6%. Pharmact AG, Germany developed SARS-CoV-2 Rapid, which utilizes two drops of blood sample from the patients and can provide results in 20 min (56). The obtained results can be correlated and confirmed with RT-PCR.

Chembio Diagnostics, USA has recently developed a DPP COVID-19 IgM/IgG POCT system that is based on the LFIA test and can provide results within 15 min using a drop of blood sample (57). This system utilizes the data readout via MicroReader 1 and 2 analysers. VITROS Immunodiagnostic Products Anti-SARS-CoV-2 Total Reagent Pack/Total calibrator was also approved by FDA for EUA; it was developed by Ortho-Clinical Diagnostics, Inc., USA based on a modified ELISA method (58). The target antigen used is S protein; this method detects both IgG and IgM but cannot distinguish between the two. Mount Sinai Laboratory, USA developed a COVID-19 ELISA IgG Antibody Test kit that utilizes ELISA on a 1:50 diluted serum flown on a plate pre-coated with S protein RBD. The IgG antibodies present in the serum bind to the antigen and are detected by the method (59).

Given that POCTs can be associated with false/positive results, further tests are required to establish their accuracy. These tests rely on the presence of antibodies in a patient, which generally develop after several days or weeks after the viral infection. Moreover, the strength of antibodies depends on the age, severity of disease, nutritional status of the patient, and ongoing medications. These antigen-detecting kits can also react with other pathogens and can be non-specific, giving false-positive results. With these reasons, the WHO discourages the use of rapid immunodiagnostic tests for patient care but encourages the development of such tests owing to their usefulness in the surveillance of disease and epidemiological studies.

### RT-PCR

RT-PCR is the most widely employed IVD test for the confirmatory detection of COVID-19. A highly specific, novel, and robust RT-PCR assay was developed by Tib-Molbiol, Germany; this assay can specifically detect SARS-CoV-2 but not other CoVs (60). The developed test can detect the RdRp gene and viral RNA E protein. The assay involving E gene gave preliminary results, whereas the RNA polymerase assay was used for confirmatory results.

Another relatively quick RT-PCR method was developed to target the Orf1b and N regions of the virus; this method provides results in a little more than an hour (61). The N-gene assay gives the initial results, and the Orf1b assay confirms the diagnosis. However, this assay can also detect other closely related sarbecoviruses, such as SARS-CoV, due to the presence of Orf1b and N regions. This problem can be overcome by using sequence analysis of positive amplicons once the RT-PCR test is positive. Another RT-PCR assay was developed targeting the RdRp and helicase genes of the virus with an added advantage of specificity for SARS-CoV-2 (62). This assay is highly sensitive and can be used specifically for COVID-19 detection despite the low viral loads. In the continuation of the development of RT-PCR assays that can yield results rapidly, a real-time rapid test (Xpert® Xpress SARS-CoV-2 test) was recently developed by Cepheid, USA (63). This test gives confirmatory results within 45 min and can qualitatively detect the virus in different specimens, such as oropharyngeal/nasopharyngeal swabs, nasal wash, or aspirates. This test has received US FDA EUA approval and targets multiple regions of the SARS-CoV-2 genome.

However, mounting evidence shows that RT-PCR methods cannot detect the virus especially in the early stages of infection, giving false negative results (64, 65). The false negative results can be attributed to the insufficient and improper extraction of nucleic acid for the test. Therefore, in these cases, a computerized tomography scan of the chest is suggested as a complementary tool (66, 67). Therefore, a suitable diagnostic assay which can accurately detect the specific biomarkers of SARS-CoV-2 in the initial stages of infection is still required.

## Development of Vaccine

Since the genetic sequence of SARS-CoV-2 came to the public domain on January 11, 2020, an intense research was triggered to develop a suitable vaccine for the virus. The development of vaccine for human use generally takes 12–18 months under unprecedented circumstances and rapidity. The clinical trial for the first vaccine candidate has already started on March 16, 2020. A Coalition for Epidemic Preparedness Innovations was established; it is continuously working with vaccine developers and health authorities globally for the development of COVID-19 vaccines. Thus far, 115 vaccine candidates from different companies have been developed. Out of these candidates, 78 have shown confirmed activity, whereas the data for 37 candidates are not available publicly or are unconfirmed. Out of the 78 confirmed candidates, 5 entered the clinical stage, whereas the remaining 73 are still at the preclinical or exploratory stage. The most advanced five candidates which entered the clinical phase include Ad5-nCoV (CanSino Biologics) (68), mRNA-1273 (Moderna) (69), pathogen-specific aAPC (70), LV-SMENP-DC (71) (Shenzhen Geno-Immune Medical Institute), and INO-4800 (Inovio) (72).

The phase I trial of Ad5-nCoV (CanSino Biologics) is a single-center, non-randomized, open-label and dose-escalating trial on healthy patients aged 18–60 years. The trial (108 participants) has started on March 16, 2020 and will end on December 30, 2020. The trial would primarily test the safety of the vaccine at three dose levels: low, middle, and high. The initial results of phase I have recently been published in Lancet (73); CanSino Biologics reported that the adenovirus-vectored vaccine was tested on 108 healthy volunteers in three dose levels, and most of them showed development of neutralizing antibodies and T-cell responses to the antigen. No serious adverse effects were reported, and 81% of the volunteers reported minor symptoms, such as pain, fever, headache, and fatigue. The second trial on mRNA-1273, which was developed by ModernaTX, Inc. and sponsored by National Institute of Allergy and Infectious Diseases (NIAID), is also in phase I trial and has enrolled 45 healthy volunteers aged 18–55 years old. This trial will assess the safety, immunogenicity and reactogenicity of mRNA-1273 vaccine candidate at different dose levels (25, 100, and 250 mcg). This trial is expected to be completed in June 2021. Preliminary results from the study revealed that this vaccine elicited binding antibodies in all 45 volunteers of the phase I trial. The results, which have not been released yet, suggest that all three doses (25, 100, and 250 mcg) led to seroconversion in all participants. However, the highest dose (250 mcg) arm showed development of grade 3 adverse reactions. The company has decided to proceed to phase II trial with 50 and 100 mcg doses (74).

The Shenzhen Geno-Immune Medical Institute developed two vaccine candidates with ongoing clinical trials. The first is the pathogen-specific aAPC, whose phase I trial is currently ongoing with 100 participants. Another vaccine by the same company, that is, LV-SMNEP-DC vaccine and antigen-specific CTLs are currently under phase I and II multicentre trials including 100 healthy and infected participants. The study will establish the safety and efficacy of the vaccine for the treatment of COVID-19. A new candidate developed by Inovio Pharmaceuticals in collaboration with CEPI (INO-4800) recently entered the clinical trial phase. This phase I trial would test the safety, tolerability and immunogenicity of the vaccine in 40 healthy participants and should be completed by April 2021.

Other clinical trials are intended for other vaccines, including a candidate developed by UK, which has planned to invest 50.7 million USD into two vaccine development research projects. This candidate vaccine (ChAdOx1 nCoV-19) was developed by Jenner Institute at Oxford University and uses a genetically engineered viral vector from chimpanzee (adenovirus) to carry the CoV antigen (S protein). The institute is currently recruiting participants (with 500 healthy volunteers aged 18–55 years old) by mid-May (75); the total number of expected participants is 1,110. Pfizer Inc. and BioNTech SE have secured approval from the German regulatory approval for a developed vaccine in late April 2020; the clinical trial will start with 200 healthy participants aged 18–55 years old (76). Other vaccine candidates were developed successfully in laboratory and have been transitioned to the preclinical stage; one of these candidates include a vaccine developed by University of Queensland, Australia in collaboration with a Dutch firm Viroclinics Xplore, which is planning to advance to the clinical phase in the third quarter of 2020 (77). Another candidate from the Saskatoon-based research lab in Canada is proceeding to the pre-clinical phase this month and is expected to move to clinical trials by fall this year (78). Johnson & Johnson and Sanofi are both working on their own vaccines. Several existing vaccines, for instance, that used for tuberculosis and polio, are also being tested in trials in the Netherlands (79) and Australia (80, 81) to determine their efficacy in the protection from COVID-19.

The preparation of vaccines employs different methods, which include the usage of live attenuated virus, inactivated viral particles, viral vectors (replicating and non-replicating), recombinant protein, virus-like-particle, nucleic acid (DNA and RNA), or peptide based candidates. The vaccine developed by Moderna Inc. (mRNA-1273) in collaboration with NIAID is based on mRNA and has the advantage of flexibility with regard to antigen manipulation and rapid development, prompting Moderna to start the trial within 2 months of identification of genetic sequence of SARS-CoV-2. Nevertheless, the vaccine using viral vectors that are based on lentiviral vector system, for instance, the two vaccines developed by Shenzhen Geno-Immune Medical Institute, offer strong immunological response and high level of antibody expression inside the body with long-term stability.

The usage of adjuvants increases the effectiveness of vaccines. Adjuvants are used with vaccines to increase the immunogenicity of the latter, which would ultimately reduce the required dose. Various companies are currently working on the development of adjuvants. Thus far, 10 developers have created their own plans. Several licensed adjuvants, such as AS03 (GlaxoSmithKline), MF59 (Seqirus), and CpG1018 (Dynavax), are planned to be used with the developed vaccine, and their effect on efficacy will be tested. Owing to the unprecedented effort by different research authorities globally in terms of scale and speed, the first vaccine for emergency human use can be available by early 2021. This event would be a major achievement given that t normal process of vaccine development takes around 10 years. For Ebola, the accelerated vaccine development virus lasted for 5 years. Several pharmaceutical giants, such as GlaxoSmithKline, Janssen, Sanofi, and Pfizer, along with other smaller companies are involved in the development of COVID-19 vaccine.

## Developments In Drug Repurposing

No antiviral treatment has been approved for COVID-19 to date, and the foremost way of treatment is still symptomatic. A number of already approved antiviral drugs are tested clinically to determine their efficacy against SARS-CoV-2 given that most viruses share similar genome. Drug repurposing is the most likely means to combat the virus at the moment given the considerable time required for other methods. A number of clinical trials have already been conducted along with several ongoing trials based on the test of already approved drugs used in COVID-19 patients. These trials are phase III trials of infected persons and study the safety and efficacy of these drugs on the patients. The approved antiviral treatment for COVID-19 is likely to be achieved before the vaccine. An extensive target list for the repurposing of pharmacological agents was prepared through a multi-collaborative effort using 26 cloned viral proteins. The list identifies 69 USFDA-approved drugs that can potentially disrupt the virus–host interaction, but it will require extensive *in vivo* validation (82).

### RdRp Inhibitors

SARS-CoV-2 is an RNA virus and thus requires the RdRp enzyme for replication. Therefore, the drugs inhibiting RdRp would result in the premature termination of viral RNA transcription. Three antiviral drugs which belong to this class have been tested for COVID-19: remdesivir, favipiravir, and ribavirin. Remdesivir (GS-5734, Gilead Sciences) is the single *Sp* isomer of 2-ethylbutyl-*L*-alaninate phosphoramidate prodrug, which was introduced a decade ago for the treatment of Ebola virus. Remdesivir resembles the RNA ATP building block in terms of structure. RdRp is incorporated in the chain, and further incorporation of RNA subunits is stopped. Remdesivir has previously shown promising activities against a number of RNA viruses, including SARS-CoV and MERS-CoV, in *in vitro* experiments and preclinical studies. This drug has also shown significant *in vitro* activity against SARS-CoV-2 (83) and is among the front runners for the drug therapy of COVID-19. As an inhibitor of viral replication, remdesivir is expected to be effective in the early stages of infection. The results of the compassionate use of remdesivir were published by Gilead Sciences, which conducted studies on 61 severely ill patients from US, Europe, Japan and Canada. When remdesivir was administered at a dose of 200 mg once on the first day followed by 100 mg once daily for 9 days to patients on mechanical ventilation, 57% of the patients were weaned-off from ventilation, 47% were discharged, and 13% died. (84). Another randomized, double-blinded, placebo controlled trial on 236 patients was conducted in 10 hospitals in Wuhan, China; the results were published recently and revealed that remdesivir failed to exhibit any significant benefit compared with the control (85). This study was planned for 453 patients earlier but was underpowered due to the unavailability of participants. However, the adaptive COVID-19 treatment trial sponsored by NIAID on 1,063 patients in the US revealed that the median recovery time in patients receiving remdesivir was reduced to 11 days compared with 15 days for the placebo (86). The still unpublished results showed 31% faster recovery time in the treatment group and a reduced mortality rate of 8% compared with 11.6% in the placebo group. Currently, six ongoing clinical trials are testing the efficacy and safety of remdesivir on moderately and severely ill patients in various parts of US, China, Japan and France (NCT04292730, NCT04292899, NCT04280705, 2020-000936-23, NCT04252664, and NCT04257656).

Another promising RdRp inhibitor, favipiravir (T-705), is a pyrazinecarboxamide drug with brand name Avigan; this drug was developed by Fujifilm, Japan, and is approved in the country for the treatment of influenza virus (87). A preliminary clinical trial conducted in February this year on 80 patients in China revealed that favipiravir can reduce the viral load and considerably improve the clinical conditions of patients compared with the protease inhibitor antiviral drugs lopinavir/ritonavir (88). Another trial in China was conducted on 340 patients to test the efficacy of favipiravir and compare the results with those of patients receiving standard care only. The arm that received favipiravir with standard care showed clearance of viral load in 4 days in comparison with the control arm receiving standard care only, which showed viral clearance in 11 days (89). The efficacy of favipiravir was again tested and compared with a viral entry inhibitor drug umifenovir (Arbidol) in China; the results showed better recovery rate and clinical outcomes for the arm receiving favipiravir on day 7. This study is published on a preprint server and is non-peer reviewed (90). Two other phase II and III trials are ongoing in USA and Japan, respectively, with a focus on a large number of patients. The trials would test the efficacy and safety of favipiravir.

Ribavirin (Bausch Health Companies, USA) is a guanosine analog used for the treatment of other viral infections, including hepatitis C virus and respiratory syncytial virus. Previously, ribavirin was a part of triple therapy, which included interferon (IFN)-α2a and lopinavir/ritonavir, for the treatment of MERS-CoV in South Korea (91). Several ongoing clinical trials are testing the efficacy and safety of ribavirin alone and in combination with protease inhibitors lopinavir/ritonavir and IFNs (92–94). β-D-N^4^-Hydroxycytidine (EIDD-1931) is a ribonucleoside analog that possesses broad-spectrum activity against difference CoVs, including SARS-CoV-2, SARS-CoV, MERS-CoV, and other related zoonotic groups such as 2b or 2c Bat-CoVs. This molecule also shows an increased potency against CoVs that are resistant to other nucleoside analog inhibitors (95).

### Protease Inhibitors

Protease or proteinase inhibitors target the papain-like and main proteases, thereby inhibiting proteolysis in viruses. These inhibitors are anti-retroviral drugs and are approved for the treatment of human immunodeficiency virus (HIV). These proteases play important roles in the processing of polyproteins and replication in viruses. When the genomic sequence of SARS-CoV-2 was compared with that of SARS-CoV, the catalytic site for protease was conserved in novel CoVs. Thus, drug re-purposing targeting this site can be plausible. The drugs falling under this category include lopinavir, ritonavir, and darunavir, which have shown promising activities against COVID-19.

Lopinavir is a peptidomimetic molecule containing hydroxyethylene scaffold, which has structural similarity to the peptide linkage targeted by protease enzyme. Given its poor oral bioavailability and extensive biotransformation, lopinavir is often prescribed with another protease inhibitor, ritonavir. Ritonavir itself lacks a good activity but binds to and inhibits the enzyme CYTP4503A, which is responsible for the metabolism of lopinavir, thereby increasing its half-life. The combination of both drugs is marketed under the brand name “Kaletra,” which was approved for the treatment of HIV infection and has previously shown good efficacy against SARS-CoV (96). However, a recent clinical trial on 199 COVID-19 patients conducted in China in January–February this year did not reveal any promising efficacy of this combination in comparison with the control arm receiving standard care only. No significant improvement in the clinical symptoms and no significant reduction in the death cases were observed for the group receiving Kaletra in comparison with the control (97). A second trial aimed to compare the combination of lopinavir/ritonavir, IFN-β, and ribavirin with the control group receiving lopinavir/ritonavir only. This study was conducted in six hospitals in Hong Kong and recruited 127 COVID-19 patients having mild symptoms. The results suggested the reduced viral shedding time from 12 days in the control group to 7 days in the treatment group receiving the combination of four agents (98). Another trial was based on the comparison of efficacy of lopinavir along with viral entry inhibitor, umifenovir (Arbidol), with the control group receiving standard care only (99). The results showed no significant benefit of lopinavir in comparison with the control. Despite the discouraging findings, the number of deaths in the drug-receiving group was slightly lower than that in the group receiving standard care only; comparatively, larger doses of the drug might be required to inhibit SARS-CoV-2 replication (91, 100). A multicentre, open label, randomized controlled trial is ongoing in China to determine the efficacy of Kaletra.

Another protease inhibitor, darunavir (Prezista), also showed promised in *in vitro* activities against SARS-CoV-2 (101) and inhibition of viral replication at 300 μM concentration. This inhibitor (Janssen Pharmaceutica, Belgium) was approved for the treatment of HIV in combination with cobicistat or ritonavir, which boosts the activity of darunavir (102). However, the company denied any evidence of activity of darunavir against COVID-19, as revealed from the clinical trials conducted on the drug in China. A small phase III clinical trial on 30 patients who received the darunavir/cobicistat combination showed no significant benefit in comparison with the control group receiving standard care only (103).

### Viral Entry Inhibitors

Viral entry inhibitors are drugs that inhibit the entry of viruses to host cells. Umifenovir (Arbidol; Pharmstandard) shows a desirable efficacy against the influenza virus and is approved for treatment in Russia and China. Chemically, this inhibitor consists of an indole scaffold which is highly substituted with different functional groups. Umifenovir prevents the fusion of viral E and cell M of target cells, thereby preventing the entry of virus into cells. Owing to its promising activities against a variety of enveloped and non-enveloped viruses, umifenovir was tested against SARS-CoV-2 *in vitro* and has shown to inhibit the virus at a concentration of 10–30 μM (104). This has led to its clinical trial which was conducted in China in February–March of this year along with favipiravir. A total of 120 COVID-19 patients were enrolled for the study, and they were divided into three groups receiving arbidol, favipiravir, and standard care. The clinical recovery rate after 7 days was assessed; the arbidol-receiving arm showed 55.86% recovery rate on day 7 in comparison with favipiravir, which showed 71.43% recovery rate (105). This result encouraged three more phase IV clinical trials for arbidol in China with the use of a larger number of samples (106–108).

### IFNs

IFNs are a group of soluble glycoproteins induced in response to specific extracellular stimuli such as viral infection. They are α-helical cytokines that are expressed through stimulation of toll-like receptors. Thus far, three classes of IFNs have been identified, namely, alpha (α), beta (β), and gamma (γ). IFNs modulate the immune system response to infection from viruses, bacteria or any foreign substances. They do not directly kill the foreign substances but instead simulate the immune system to combat infection. Commercially available IFNs acting as drug substances are prepared using recombinant DNA technology, and many types of IFNs have been developed and used to treat various conditions. IFN β-1a, IFN α-2a, and IFN α-2b are under investigation for their potential against COVID-19. IFN β-1a activates macrophages, which can engulf viral antigens, and natural killer T- cells, which are released from the thymus. The disadvantage with the use of IFNs is their capability to worsen the flu-like symptoms of COVID-19 to flare up the immune system. Therefore, their use in severely ill patients with chronic symptoms is avoided and should only be used as a last resort.

IFN-α alone and in combination with antiviral drugs ribavirin and Kaletra has shown efficacy against COVID-19 (109). IFN-α2a was also part of the triple therapy used for MERS-CoV infection. The sensitivity of SARS-CoV-2 is higher for IFNs in comparison with SARS-CoV; the inhalation of IFN-α2b decreased the infection rate considerably and can be utilized in the prophylaxis of COVID-19 (110, 111). Moreover, the infection from SARS-CoV-2 leads to the suppression of IFN-β production, which provides protection to the immune system. Recently, a UK-based biotechnology company, Synairgen, received approval to conduct trials with IFN-β on COVID-19 patients (112).

### Monoclonal and Polyclonal Antibodies

Monoclonal and polyclonal antibodies have been used earlier as therapeutic and prophylactic tools against viral infections including influenza virus. However, *in vivo* studies suggested that the protection provided by these antibodies is effective only in the early stages of infection and not in severely ill patients (113). Earlier, the safety of a polyclonal antibody SAB-301, which is produced in transchromosomic cattle, was assessed in a phase I trial, in which the dose of up to 50 mg/kg was found to be safe (114).

Two monoclonal antibodies, namely, sarilumab, and tocilizumab, are being tested for their efficacy and safety in COVID-19 patients. Sarilumab (Kevzara) is a human monoclonal antibody that is expressed against the lung inflammation induced by interleukin-6 (IL-6). This antibody was invented by Regeneron Pharmaceuticals, USA, which has now collaborated with another pharma giant Sanofi to conduct phase II and III trials to evaluate the efficacy of sarilumab in around 400 COVID-19 patients. Lung inflammation is one of the major symptoms of COVID-19; thus, this antibody is expected to attenuate the inflammation by blocking the IL-6 receptors (115, 116). Regeneron Pharmaceuticals has also collaborated with Gilead Biosciences and Feinstein Institute to conduct a trial on the concomitant use of sarilumab with remdesivir (117). Recently, Regeneron has developed an investigational dual antibody cocktail named REGN-COV-2 for the prevention and treatment of the disease and started a phase I trial to evaluate its safety and efficacy in hospitalized and non-hospitalized patients with COVID-19 (118).

Tocilizumab (Actemra) was developed by Roche Pharmaceuticals, Switzerland, and it was approved for the treatment of exacerbation of rheumatoid arthritis. This antibody is also specific for IL-6 receptor. A study conducted on moderately, severely and critically ill 15 COVID-19 patients in China has been published recently. Tocilizumab was given alone and in combination with methylprednisolone; it decreased the C-reactive protein level rapidly but caused a dramatic increase in IL-6 level in several patients (119). Currently, tocilizumab is being tested in as many as 24 clinical trials on COVID-19 patients worldwide; several early reports indicated promising immunomodulatory activity (120). Recently, labeled SARS-CoV-2 S protein RBD was used as a probe to sort antigen-specific B-cells from COVID-19 patients, and 206 monoclonal antibodies that can bind to RBD were developed (121).

### Quinoline Derivatives

Two quinoline derivatives, chloroquine (CQ) and its hydroxy derivative hydroxychloroquine (HCQ), have shown promising potential in the treatment of COVID-19. Although, mixed results are observed with the therapy, they are still considered good candidates for SARS-CoV-2 infection, and the results of ongoing trials are awaited. According to an *in vitro* study carried out in China, CQ has shown significant inhibitory activity against the virus, showing the EC_50_ value of 1.13 μM (83). As many as 20 clinical trials are being conducted to check the safety and efficacy of CQ and HCQ on COVID-19 patients.

A recently conducted trial using CQ on 100 COVID-19 patients showed improvement in lung images and pneumonia along with the shortening of duration of treatment compared with the control receiving standard care (122). However, another trial conducted in Brazil revealed discouraging results when they used two doses of CQ, namely, a low (2.7 g over 5 days) and a high dose (12 g over 10 days), on COVID-19 patients. The trial conducted in Manaus Public Hospital in Brazil reported that the high-dose CQ group patients showed increased number of deaths which led to the halting of treatment for this group (123).

HCQ, which is less toxic than CQ, was also used on COVID-19 patients. Mixed results were obtained from different trials. An earlier published study in France revealed good efficacy of HCQ when given alone and in further improvement of the clinical conditions and reduction in the duration of therapy when given in combination with the antibiotic azithromycin (124). However, discouraging HCQ trial results on came from another study in France, where the group receiving HCQ was dropped owing to the increased cardiovascular complications in this arm. The patients of this group showed abnormal prolongation of QTc interval and heart rhythm (125). Another similar study involving HCQ and azithromycin showed non-significant results and negligible improvement in the clinical conditions of COVID-19 patients receiving the combination in comparison with the control with increased risk of cardiovascular complications (126). HCQ was tested for its prophylactic use; a study carried out at multiple centers in USA and Canada found no benefit of the drug in decreasing the incidence of the disease (127). The trial was performed on 821 asymptomatic people who were at high risk of viral exposure and were predominantly healthcare workers. A total of 107 participants developed the disease, and no significant difference was observed between the treated (49 out of 414) and placebo (58 out of 404) groups. A retrospective study on HCQ and CQ used more than 96,000 patients, and the results were published in Lancet (128); the data collected by Surgisphere Corporation showed that the drugs offer no significant benefit either alone or in combination with azithromycin. This finding led to a temporary pause in the clinical trials on CQ and HCQ worldwide. After an open letter from many researchers questioning the validity of the data, the article was retracted.

Results of an observational study of HCQ use in hospitalized COVID-19 patients were published recently; in the study, the association between the use of HCQ and intubation or death was studied at a medical center in New York (129). Out of the 1,376 patients, 811 received HCQ within 24–48 h of presentation to the emergency department. Overall, 346 patients had a primary end point and were either intubated or died, and no significant association of HCQ administration was observed with the lowered or increased incidence of intubation or death. Similarly, an unpublished RECOVERY COVID-19 trial conducted by the University of Oxford, UK resulted in the suspension of HCQ arm (130). The trial included 1,542 patients receiving HCQ and 3,132 patients receiving standard care. No significant effect was noted on the death rate or the duration of hospital stay. Owing to the inefficacy of HCQ and CQ in various clinical trials, FDA has recently revoked the EUA of both drugs and stated that the potential benefits of these drugs in treating COVID-19 do not outweigh their known adverse effects (131).

### Convalescent Plasma

Convalescent plasma is obtained from patients who have recovered from COVID-19; it generally contains good concentration of antibodies produced by the body in response to the infection. This method has been used successfully in the past for the treatment and management of SARS-CoV, MERS-CoV, and Ebola virus (132–135). Convalescent plasma is regulated as an investigational product because it lacks approval by FDA. Convalescent plasma showed positive results in a study conducted between January–February of this year in China, where the recovered patients with high antibody titer values were selected for plasma collection. Transfusion of 200 mL plasma to 10 critically ill patients resulted in rapid improvement in clinical conditions within 3 days of infusion in half of the patients (136). Another study involving the transfer of convalescent plasma was conducted, in which the plasma of five donors who recovered from COVID-19 and had high titres of IgG antibodies were given to five patients on mechanical ventilation. Interestingly, three of the five patients were weaned from the ventilation and were discharged (137). However, a study recently published in JAMA (138) reported the results of a multicentre randomized control trial carried out in seven medical centers in Wuhan City, China. The study enrolled 103 participants with severe and life-threatening COVID-19, out of which 52 received convalescent plasma therapy. No significant clinical benefits were reported although the study was terminated early and was underpowered due to the lack of patients in the city. Nevertheless, this therapy suffers from several limitations, and it is also associated with the risk of transmission of other diseases. Moreover, the antibodies should be present in high titer value in the patients from which the plasma can be obtained.

### Herbal Drugs

Although the clinical evidence for herbal drugs is scarce, several traditional Chinese medicines, and herbal formulas have shown activity against SARS and H_1_N_1_ viruses. In the systemic analysis of historical records and evidence from different data sources, several herbal medicines which could have promising activities against SARS-CoV-2 were identified. A total of 28 traditional medicines that can provide treatment for COVID-19 were identified; out of these traditional medicines, 26 were of government-issued Chinese guidelines, and 2 were issued by Korean medicine professional associations (139). According to the Chinese guidelines, herbal drugs, Glycyrrhizae Radix et Rhizoma (Gancao), Astragali Radix (Huanggi), Saposhnikoviae Radix (Fangfeng), Lonicerae Japonicae Flos, Macrocephalae Rhizoma (Baizhu), Fructus forsythia (Lianqiao), Armeniacae Semen Amarum, Gypsum Fibrosum, and Ephedra Herba have been frequently used for the symptomatic and antiviral treatments and can be tested for COVID-19. However, extensive clinical trials are required to establish the efficacy and safety of these traditional Chinese medicines on humans (140, 141).

### Corticosteroids

The RECOVERY group at Oxford University, UK has recently issued a statement regarding the use of corticosteroid dexamethasone, which reduces the risk of death in severely ill COVID-19 patients requiring oxygen support (142). The randomized trial included low-dose dexamethasone (6 mg) either given orally or intravenously to 2,104 COVID-19 patients for 10 days. The results were compared with the control arm, which included 4,321 patients who were given standard care only. The mortality rate in the case of patients on mechanical ventilation reduced to one-third in the dexamethasone arm compared with the 41% observed in the control group. The death rate reduced by 20% in patients receiving supplemental oxygen support, whereas no effect was noted in patients requiring no oxygen support.

## Conclusions

The SARS-CoV-2 pandemic, which is growing rampant across the borders, is a frightening global concern and is currently the most important health emergency around the world. The lack of vaccine and a suitable treatment for the disease further worsens the issue. The ongoing research relies on rapid and accurate diagnostic techniques, vaccine development and identification of effective therapy out of the existing drugs. Early diagnosis of infected individuals is the most important step, and a suitable diagnostic technique that can accurately detect the virus in the early stages of infection is sought after. Several POC techniques developed by healthcare agencies have shown promising results in detecting the virus rapidly and accurately. Abbott ID Now^TM^ can detect the virus in 5 min. Similarly, the number of vaccine candidates has already been determined by various laboratories and pharmaceutical companies, several of which have entered the clinical trial phase. The development of a suitable vaccine would be a great achievement and is perhaps the best hope for ending this pandemic. The potential approaches to developing COVID-19 vaccine are identified from the work conducted on previously known viruses, such as SARS-CoV and MERS-CoV. The clinical trials carried out on the already approved drugs for their repurposing for COVID-19 yielded mixed results. Several antiviral drugs, such as remdesivir and favipiravir, have shown promising results in reducing the viral load and the duration of therapy. However, more evidence is needed for these antiviral drugs to be established as therapy for COVID-19. New multicentre clinical trials on large number of patients are ongoing around the world to gain insights into the development of a suitable vaccine and treatment for COVID-19.

## Author Contributions

WA, KP, BM, and SJ collected the data and wrote the manuscript. HA, MA, AN, MS, and HM collected the data, conceptualized and designed the work, and reviewed the manuscript. AK, SM, MT, and SS collected the data, helped in writing the manuscript, and proofread and critically reviewed the manuscript. All authors contributed to the article and approved the submitted version.

## Conflict of Interest

The authors declare that the research was conducted in the absence of any commercial or financial relationships that could be construed as a potential conflict of interest.
